# Simultaneous targeting of mitochondrial metabolism and immune checkpoints as a new strategy for renal cancer therapy

**DOI:** 10.1002/ctm2.645

**Published:** 2022-03-29

**Authors:** Sona Stemberkova‐Hubackova, Renata Zobalova, Maria Dubisova, Jana Smigova, Sarka Dvorakova, Klara Korinkova, Zuzana Ezrova, Berwini Endaya, Kristyna Blazkova, Erik Vlcak, Petra Brisudova, Dan‐Diem Thi Le, Stefan Juhas, Daniel Rosel, Cristina Daniela Kelemen, Dana Sovilj, Eliska Vacurova, Tomas Cajka, Vlada Filimonenko, Lanfeng Dong, Ladislav Andera, Pavel Hozak, Jan Brabek, Zuzana Bielcikova, Jan Stursa, Lukas Werner, Jiri Neuzil

**Affiliations:** ^1^ Institute of Biotechnology Czech Academy of Sciences Prague‐West Czech Republic; ^2^ Faculty of Science Charles University Prague 1 Czech Republic; ^3^ School of Pharmacy and Medical Science Griffith University Southport Qld Australia; ^4^ Institute of Molecular Genetics Czech Academy of Sciences Prague 4 Czech Republic; ^5^ Institute of Animal Physiology and Genetics Czech Academy of Science, PIGMOD Centre Libechov Czech Republic; ^6^ Institute of Physiology Czech Academy of Sciences Prague 4 Czech Republic; ^7^ General University Hospital Prague 1 Czech Republic

Dear Editor,

Cancer is a pathology still on the rise,^1^ with unmet need for efficient therapy, owing to factors such as considerable differences in mutational signature in the same patient in primary tumours and proximal/distal metastases, shown, for example, for renal cancer.^2^ What is needed then is an invariant target predominantly only affected by drugs in cancer cells. A thus far untested approach is targeting mitochondrial respiration using compounds from the group of mitocans,^3^ epitomised by mitochondrially targeted tamoxifen (MitoTam), that is, tamoxifen tagged with the mitochondrial vector triphenylphosphonium (TPP) (Figure S1A; see also Supporting Information for description of synthesis).^4^, ^5^ This strategy is based on the premise that cancer cells differ from their non‐cancerous counterparts,^3^ making them selectively vulnerable to TPP‐tagged anti‐cancer agents,^6^ and on the premise that mitochondrial function is vital for tumour progression.^7^, ^8^

We have recently conducted Phase 1/1b MitoTam clinical trial for metastatic solid tumour patients, with all patients undergoing palliative therapy after exhaustion of established therapeutic regimens (MitoTam‐01 trial; EudraCT 2017‐004441‐25). Although the Phase 1/1b clinical trial will be published in its entirety elsewhere, of the individual types of cancer, the greatest benefit was found for clear cell renal cancer patients represented here by two subjects (Tables S1 and S2). These patients underwent three and four rounds of MitoTam therapy, respectively, at 1 mg/kg three times per week followed by a week of rest, totalling four such cycles, with one patient showing tumour stabilisation and the other partial remission (Figure 1A). The trial revealed excellent safety profile of MitoTam, with only occasional grade 1 toxicity. The high efficiency for renal cancer was found to correlate with the highest level of MitoTam and its metabolites reached in kidneys (Figures 1B, S1B and S1C), being excreted via bile (Figure S1D).

**FIGURE 1:**
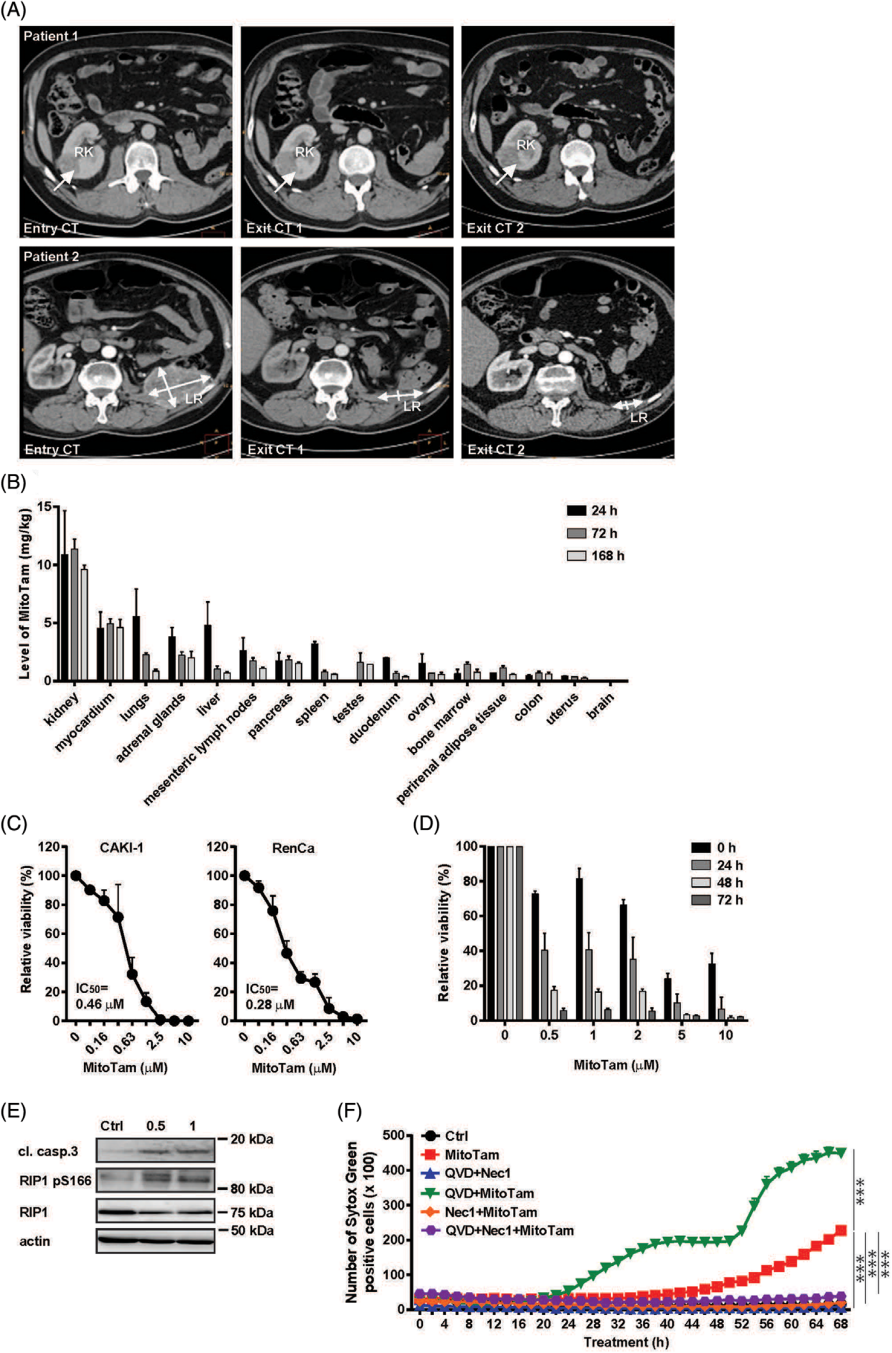
MitoTam is efficient against renal cancer in patients, accumulates in kidney and kills renal cancer cells. (A) CT scans are presented for two renal cancer patients from Phase 1b clinical trial. Patient 1 showed stabilisation of target lesion in right kidney that formed after left nephrectomy to remove primary tumour in left kidney. Patient 2 experienced partial remission of relapse in surgical bed after left nephrectomy with primary tumour, showing 30‐fold reduction in the tumour volume. The exit CT scans document the size of the tumour after cycles 2 (CT2 and 3 (CT2) of therapy. (B) Distribution of MitoTam was determined in pigs. The drug was administered via ear vein at 6 mg/kg. MitoTam content was analysed by liquid chromatography‐mass spectroscopy (LC‐MS) in selected tissues 24, 76 and 168 h after administration. (C) Renal cancer cell lines were treated with MitoTam for 24 h as shown and viability was assessed by the Crystal violet assay. (D) and (E) RenCa cells were treated with 0.5 or 1 μM MitoTam for 24 h, and cleaved caspase‐3 and RIP1 (total and phosphorylated on S166) were analysed by western blotting (WB). Actin was used as loading control. (F) RenCa cells were seeded in a 96‐well plate at 10^4^ per well and treated with 0.5 μM MitoTam in the presence of the pan‐caspase inhibitor Q‐VD‐OPh (QVD) at 50 μM or the necroptosis inhibitor necrostatin‐1 (Nec1) at 50 μM, and assessed for viability using Syto‐Green and the Lumasccope LS720 scanning microscope. Data are mean values from three independent experiments ± SEM

As renal cancer cells possess high activity of oxidative phosphorylation (OXPHOS) and its inhibition induces cell death in starvation‐resistant tumours,^7^ we studied the effect of MitoTam on renal cancer in vitro and in vivo to better understand its benefit for patients. Using human and a mouse renal cancer cell lines, we found that MitoTam killed the cells with IC_50_ of ≈0.3–1.4 μM (Figure 1C, Figure S1E) even after its removal from cell culture media (Figure 1D). Analysing the mode of action using RenCa cells, we found necroptosis to play major role in elimination of cancer cells (Figures 1E and F, Figure S2A), while inhibition of caspases enhanced the MitoTam effect (Figure S2B). This indicates positive connotation for inhibiting renal cancer by killing cancer cells via multimodal manner.

Importance of mitochondria for killing cancer cells by MitoTam was tested using cells with modified mitochondrial function^8^ (Figure S2C–S2E). Consistent with previous findings for breast cancer cells,^5^ MitoTam inhibited primarily CI‐dependent respiration in RenCa cells (Figure 2A and B), which was accompanied by uncoupled respiration (Figure 2C), a drop in mitochondrial potential and a switch to glycolysis (Figure S2F–S2K). This was further supported by a drop in the respirasome level and CI‐ and ATPase‐dependent activity upon MitoTam treatment (Figure 2D and E) as well as in levels of CI‐CIV subunits (Figure 2F), in deregulation of the Krebs cycle and amino acid metabolism (Figure 2G and H, Table S3) and in alteration of mitochondrial morphology (Figure S2L).

**FIGURE 2:**
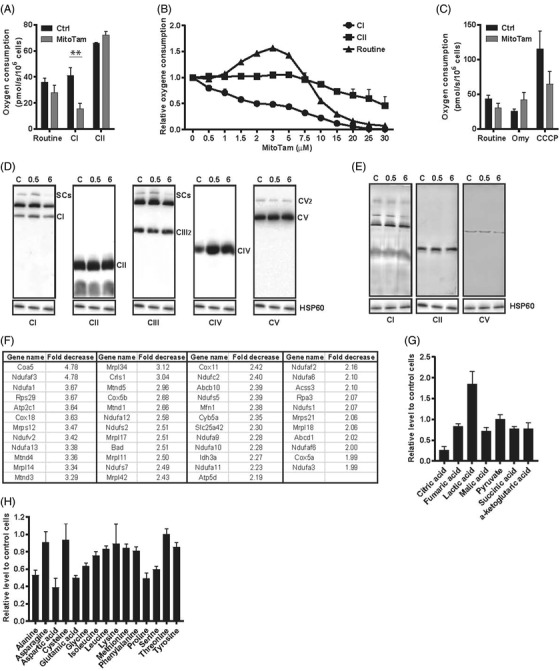
MitoTam affects mitochondrial function and metabolism. (A) RenCa cells were treated with 0.5 μM MitoTam for 6 h, and assessed for routine and CI‐ and CII‐dependent respiration using the high‐resolution respirometer Oxygraph 2k (O2k). (B) RenCa cells were placed in the O2k (10^6^ per mL), supplemented with increasing concentration of MitoTam as shown and assessed for routine, CI‐ and CII‐dependent respiration. (C) RenCa cells were exposed to 0.5 μM MitoTam for 6 h, and uncoupled respiration was assessed after addition of 1 μM oligomycin (Omy) and 1 μM CCCP in the O2k. (D) Mitochondrial fraction of RenCa cells treated with 0.5 μM MitoTam for 0.5 or 6 h was used to assess mitochondrial complexes and supercomplexes (SCs) by native blue gel electrophoresis/WB using the following antibodies: CI, NDUFA9; CII, SDHA; CIII, UQCRC2; CIV, COX5A; CV, ATPB. HSP60 was used as loading control. (E) RenCa cells were treated with 0.5 μM MitoTam for 0.5 and 6 h and evaluated for the activity of CI, CII and CV using specific in‐gel assays. RenCa cells were treated with 0.5 μM MitoTam for 6 h and assessed by LC‐MS for changes in mitochondrial protein expression (the numbers show relative decrease of protein level comparing MitoTam‐treated and control cells) (F), for metabolites linked to Krebs cycle (G) and to amino acid metabolism (H). Data are mean values from three independent experiments ± SEM

Using syngeneic model of RenCa cell‐derived tumours, MitoTam showed anti‐cancer activity in a concentration‐dependent manner (Figure 3A–C), without any effect on healthy kidneys (Figure S3A and S3B) when suppressing orthotopic renal tumours (Figure 3D and E). A similar effect was observed for CAKI‐1 cells in a xenograft model (Figure 3F and G). Although MitoTam did not inhibit proliferation of cells in syngeneic tumours, it promoted their death (Figure S3C and 3D). Selective toxicity for cancer cells was evident from suppression of CI‐dependent respiration only in renal tumours (Figure 3H–J) compared to healthy tissue without any effect on mouse weight and blood count (Figure S3E–S3F). Interestingly, MitoTam suppressed metastases of RenCa cells into lungs (Figure 3K), depending on functional mitochondria (Figure 3L). The anti‐metastatic effect of MitoTam is supported by suppression of migration and invasion of renal cancer cells in vitro using three‐ and two‐dimensional settings (Figure S3G–S3I).

**FIGURE 3:**
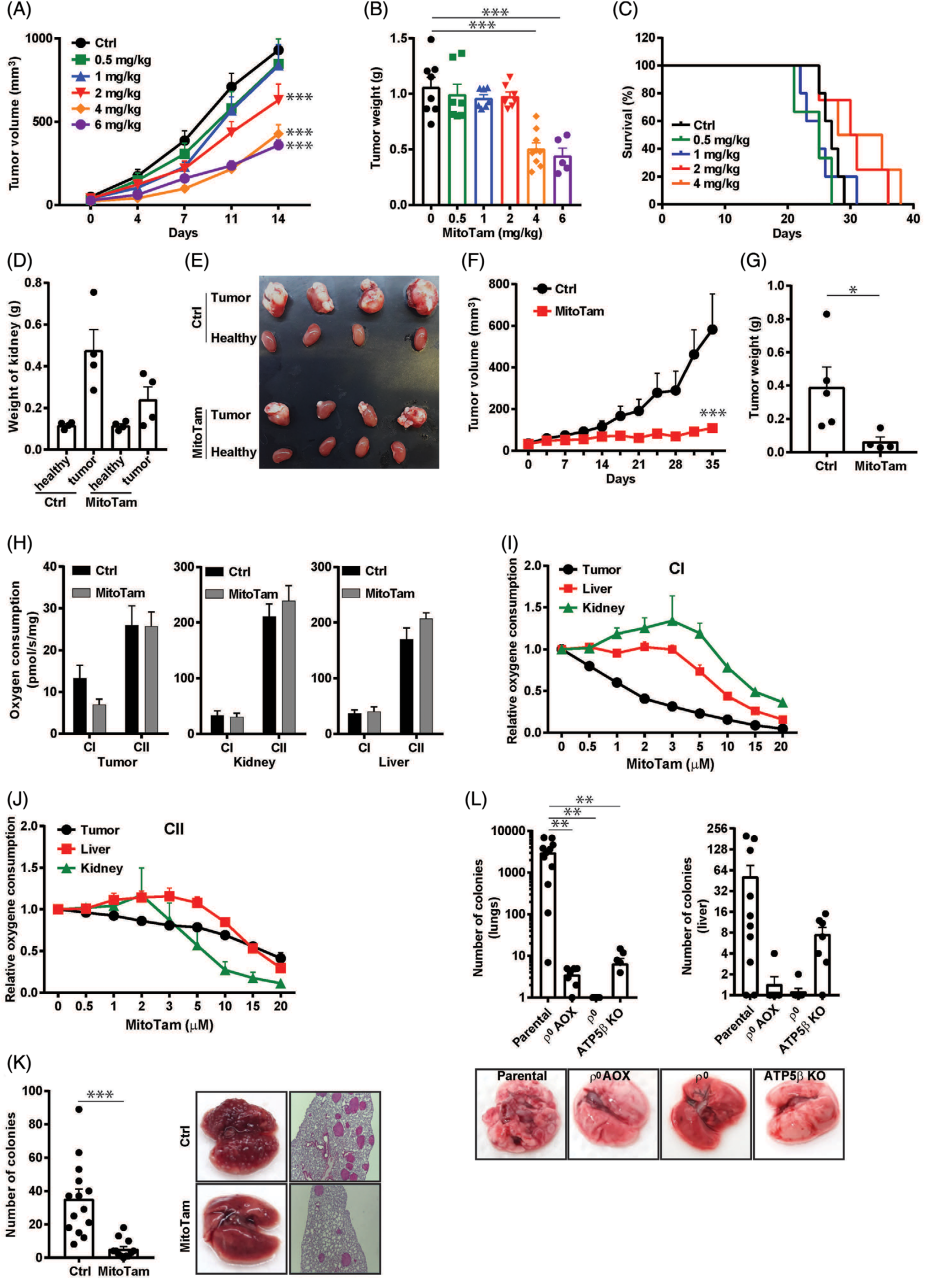
MitoTam suppresses renal tumours and metastasis by affecting mitochondrial function. (A) RenCa cells were grafted s.c. in Balb‐c mice at 3 × 10^5^ cells per animal. When small tumours appeared (∼50 mm^3^), the animals were treated by i.p. injection of MitoTam dissolved in 4% EtOH in corn oil at the time points and concentrations shown, and tumour volume was estimated as detailed in Materials and Methods. (B) At endpoint, tumours were excised and weighed. (C) Survival of mice with tumours treated with different concentrations of MitoTam was calculated as detailed in Materials and Methods. (D) Orthotopic tumours were generated by surgical grafting of RenCa cells in left kidney of Balb‐c mice (5 × 10^4^ cells per animal), and the animals treated i.p. with MitoTam at 4 mg/kg twice per week for 2 weeks. The animals were sacrificed at endpoint, and the kidney with tumour and healthy kidney were excised, weighed and (E) photographed. (F) NSG mice were grafted s.c. with CAKI‐1 cells at 10^6^ per animal and, when small tumours appeared (≈50 mm^3^), the animals were treated i.p. with MitoTam at 4 mg/kg twice per week and tumour volume was evaluated; at endpoint, tumours were excised and weighed (G). (H) Balb‐c mice grafted s.c. with 3 × 10^5^ RenCa cells per animal were treated with 4 mg/kg of MitoTam twice per week for 2 weeks. At endpoint, mice were sacrificed, and the tumour, kidney and liver were excised and assessed for CI‐ and CII‐dependent respiration using the Oxygraph . Balb‐c mice were grafted s.c. with RenCa cells at 3 × 10^5^ per animal. When tumours reached ≈500 mm^3^, the tumour, liver and kidney were excised and assessed for CI‐ (I) and CII‐dependent respiration (J) in the Oxygraph after addition of increasing doses of MitoTam. (K) Balb‐c mice were injected i.v. with RenCa cells at 10^5^ per animal and treated by i.p. injection of MitoTam at 4 mg/kg twice per week. After 2 weeks, mice were sacrificed, and lungs were excised and stained with H&E. Number of metastatic loci was evaluated under light microscope (right panel). The panels on the right show lungs excised from control and MitoTam‐treated animals and the respective cross sections. (L) Balb‐c mice were injected i.v. with parental, mtDNA‐depleted (ρ^0^) cells, ρ° cells transfected with alternative oxidase (AOX) featuring CIII and CIV function and cells with knocked our subunit of CV, ATP5B^KO^, at 10^5^ per animal. After 17 days, mice were sacrificed, and lungs and liver were excised and processed as described in Material and Methods. Colonies were stained by crystal violet and counted. The bottom panels show representative lungs from mice grafted with the four different sublines . Data are mean values ± SEM

Renal cell carcinoma is an immunogenic tumour featuring abundant infiltration of lymphocytes. Despite promising results of immunotherapy, resistance to this treatment occurs in several tumour types providing an opportunity for improved immunotherapeutic approaches. Since targeting of immune checkpoints (ICIs) PD‐L1 and PD‐1, currently representing first‐line therapy in renal cancer,^9^ induces metabolic reprogramming of cancer cells by decreased ability to use glycolysis and increased OXPHOS importance,^10^ we combined their effect with MitoTam. Based on testing different PD‐L1 concentrations on tumour growth and CD8+ T‐cell activity and recruitment (Figure S4A–S4D), we document additive effect of MitoTam and PD‐L1 used at 2 mg/kg and 400 μg/mouse, respectively, compared to single agent therapy (Figure 4A), supported by significantly greater survival (Figure 4B) and increased level of tumour cell death (Figure 4C). PD‐L1 treatment enhanced infiltration and activity of CD8+ T‐cells, which was not affected by MitoTam (Figure 4D–F). Moreover, MitoTam affected the level of PD‐L1 neither in tumours of mice with syngeneic carcinomas (Figure 4G) nor in the two cancer patients presented in Figure 1A (Figure S4E). PD‐1 showed an additive affect with MitoTam in RenCa cell tumours (Figure 4H and I) similar to that for the combination of MitoTam with PD‐L1 presented above.

**FIGURE 4:**
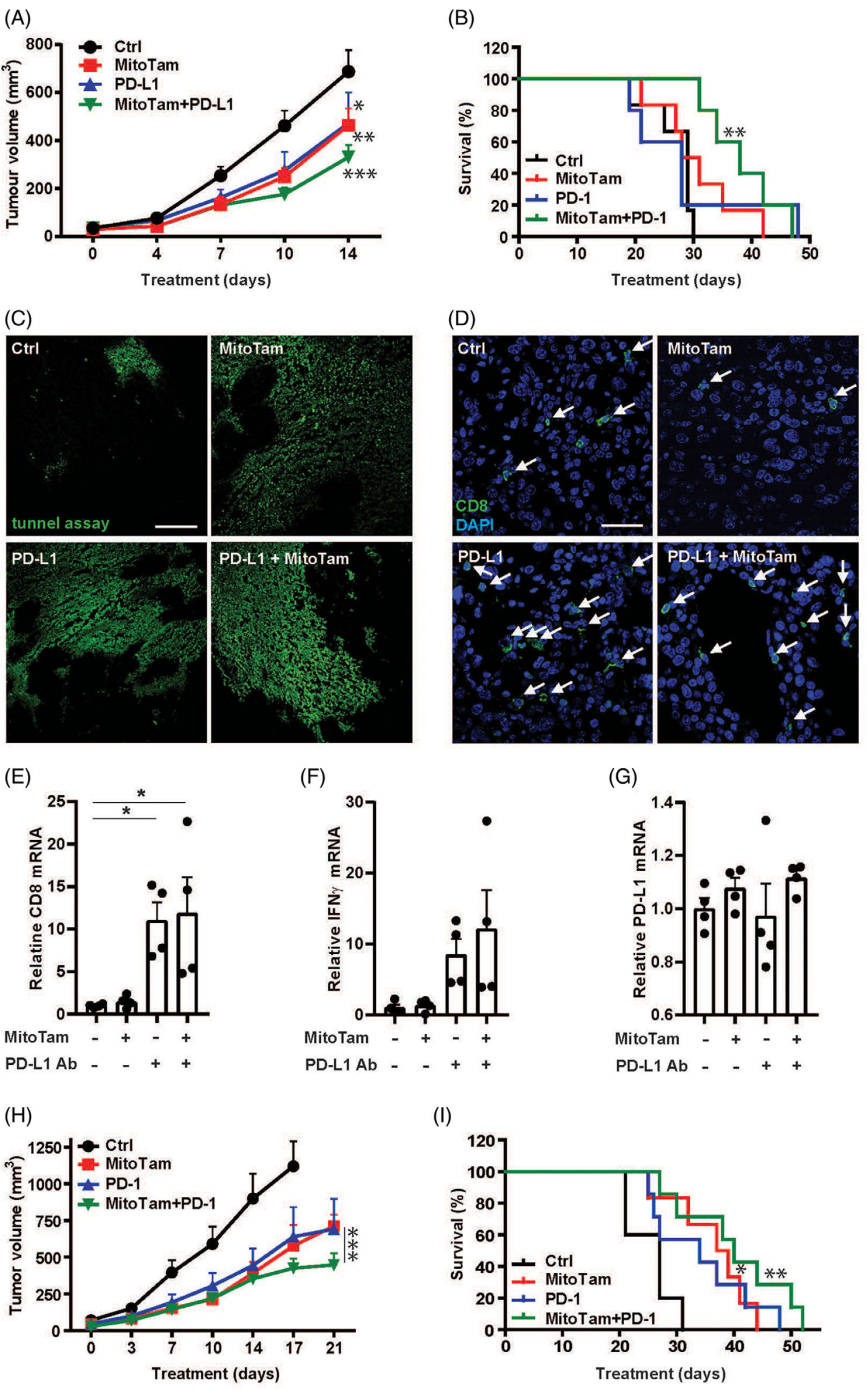
MitoTam shows combinatorial effect on renal tumours with ICIs. Balb‐c mice were grafted s.c. with 3 × 10^5^ RenCa cells. As soon as small tumours appeared (≈50 mm^3^), animals were treated i.p. with MitoTam at 2 mg/kg or anti‐PD‐L1 IgG at 400 μg per animal, or with the two agents simultaneously, as indicated. Mice were evaluated for tumour volume (A) and for survival (B). At endpoint shown in Panel A, mice were sacrificed, and sections of tumours from control mice and from animals treated with MitoTam and anti‐PD‐L1 IgG separately or in combination were stained for TUNEL‐positive cells (C) or CD8+ T‐cells (D); the scale bar represents 100 and 50 μm, respectively. Tumour tissues were assessed for levels of CD8 mRNA (E), IFNγ mRNA (F) or PD‐L1 mRNA (G) using qRT‐PCR. Balb‐c mice were grafted s.c. with 3 × 10^5^ RenCa cells. As soon as small tumours appeared (≈50 mm^3^), animals were treated with MitoTam at 2 mg/kg or anti‐PD‐1 IgG at 400 μg/mouse, or with the two agents simultaneously, as indicated. Mice were evaluated for tumour volume (H) and for survival (I). Data are mean values ± SEM. Images are representative of sections from three independent tumours per condition

To conclude, we document that MitoTam efficiently kills a range of renal cancer cells and suppresses renal carcinomas in a mouse model by a complex effect on mitochondria, resulting in decreased tumorigenesis. The fact that monotherapy of MitoTam proved to be as efficient in our animal model as immunotherapy with ICIs is highly encouraging, especially given the fact that renal cancer patients respond poorly to standard chemotherapy. Our study therefore suggests a novel strategy for treatment of renal cancer by targeting mitochondria in combination with immunotherapy presented by ICIs. As MitoTam as well as clinically approved ICIs show excellent safety profiles, these results have direct clinical implications in designing rational combination treatments and, together with Phase1/1b results, encourage further development of MitoTam in future Phase 2 trial.

## CONFLICT OF INTEREST

JN, JSt and LW are co‐owners of MitoTax s.r.o. and the MitoTam intellectual property. JN, JSt and LW are also co‐inventors of MitoTam as an oncolytic agent. Other authors declare no conflict of interest. Private investors KKCG a.s. and SmartBrain s.r.o. (both Prague, Czech Republic) financed the MitoTam‐01 clinical trial.

## Supporting information

Supporting Information

Figure S1

Figure S2

Figure S3

Figure S4

Table S1

Table S2

Table S3
