# Supplementary material for: Simultaneous targeting of mitochondrial metabolism and immune checkpoints as a new strategy for renal cancer therapy
Source: Clin Transl Med. 2022 Mar 29;12(3):e645. doi: 10.1002/ctm2.645 (PMC8964933; doi:10.1002/ctm2.645)
Supplement: Supplementary file 1 — Supporting Information [file CTM2-12-e645-s003.docx]

**MATERIAL AND METHODS**

**MitoTam synthesis**

MitoTam was synthesized in eight chemical steps. The key reaction intermediate was prepared by alkylation of iodophenol (**1**) with 2-chloro-*N,N*-dimethylethylamine hydrochloride to yield the intermediate **3**. The tertrasubstituted olephinic moiety was constructed *via* stereoselective three component palladium-catalyzed cross-coupling^1^ of 3-(4-(2-(dimethylamino)ethoxy)phenyl)prop-2-yn-1-ol (**3**) with phenymagnesiumchloride and bromobenzene. Resulting hydroxy-tamoxifen (**4**) was selectively oxidized with stabilized 2-iodoxybenzoic acid to aldehyde (**5**). Subsequent Wittig reaction with (9-hydroxynonyl)triphenylphosphonium bromide resulted in alkenylated tamoxifene (**6**). Regiospecific catalytic hydrogenation of less hindered disubstituted double bond gave single isomer of C10-tamoxifene (**7**) with a 10 carbon linker. The crucial structural element of MitoTam (the phosphonium moiety) was introduced in the last two chemical steps in a one-pot arrangement. First was the transformation of alcohol to reactive bromide (**8**) with subsequent alkylation of triphenylphosphine. The final column chromatography and ion exchange of salt (**9**) yielded +99% pure MitoTam (**10**) with overall 30% yield.

**Cell lines**

RenCa mouse renal cancer cells (derived from primary renal adenocarcinoma) were obtained from the ATCC (Manassas, VA, USA), human renal cancer cell lines CAKI-1 (derived from clear cell renal cancer skin metastasis), CAKI-2 (derived from primary clear cell renal carcinoma), 786-0 (derived from primary clear cell renal adenocarcinoma) and ACHN (derived from renal cancer pleural effusion metastatic site) were sourced from ATCC (Manassas, VA, USA). The RenCa cells were maintained in the RPMI medium supplemented with 10 % foetal bovine serum (Gibco, Carlsbad, CA, USA), 4.5 g/L glucose (Biochrom, Berlin, Germany), pyruvate (1 mM) and 100 U/mL penicillin and 100 μg/mL streptomycin sulfate (Sigma). The CAKI-1 and CAKI-2 cells were maintained in the McCoy´s 5a medium supplemented with 10% foetal bovine serum (Gibco), 4.5 g/L glucose (Biochrom) and 100 U/mL penicillin and 100 μg/mL streptomycin sulfate (Sigma). The ACHN cells were maintained in the DMEM medium supplemented with 10% foetal bovine serum (Gibco), 4.5 g/L glucose (Biochrom) and 100 U/mL penicillin and 100 μg/mL streptomycin sulfate (Sigma). The 786-0 cells were maintained in the RPMI medium supplemented with 10 % foetal bovine serum (Gibco), 4.5 g/L glucose (Biochrom) and 100 U/mL penicillin and 100 μg/mL streptomycin sulfate (Sigma). The mouse breast cancer cells 4T1 and their mtDNA-devoid variants (4T1 ρ^0^ cells) as well as mtDNA-devoid cells transfected with alternative oxidase (4T1 ρ^0^ AOX cells) and 4T1 cells with dysfunctional mitochondrial complex V (4T1 ATP5B^KO^ cells) (all cell lines were described before^2^) were maintained in the same medium as RenCa cells supplemented with 50 μg/L uridine and 5 mM pyruvate, which were removed in experiments.

**Assessment of cell viability**

The medium containing dead cells was collected into clear tube, adherent cells were trypsinized, re-suspended in the medium containing dead cells and centrifuged at 1,000 x g for 3 min. The pellet was resuspended in 200 μL of annexin V binding buffer containing 0.3 µL of annexin V-Dyomics 647 (Apronex, Vestec, Czech Republic), and incubated for 20 min at 4 °C. Hoechst 33258 (5 μg/mL) was added before the cells were analyzed using the LSR Fortessa flow cytometer. Cell viability was expressed as the percent of the annexin V-negative/Hoechst-negative fraction.

To discern the kinetics and mode of cell death, RenCa cells were seeded in a 96-well plate at 10^4^ per well and treated with MitoTam alone or in the presence of the pan-caspase inhibitor Q-VD-OPh (50 μM) and/or necroptosis inhibitor necrostatin-1 (50 μM) (both from Merck) for up to 72 h in the Lumascope LS720 scanning microscope (Etaluma, Carlsbad, CA). The LS720 instrument was placed in a CO_2_ incubator at 37 ºC, and cells were scanned for green fluorescence every 2 h. The inhibitors and Sytox-Green (ThermoFisher) were added to cells 1 h before MitoTam. Data were evaluated using the Lumaquant software (Etaluma).

**Assessment of cell proliferation**

The effect of the tested compounds on cell proliferation was assessed by the Crystal violet assay. Cell were exposed to increasing doses of the agents for different time points (see Figure legends). Cells were fixed with 4% paraformaldehyde for 15 min, stained with crystal violet (0.05% in water) for 1 h, and washed. Crystal violet dye was extracted with 100 μL of 1 % SDS and absorbance was determined at 595 nm. To obtain IC_50_ values, dose-response curves were fitted with the four-parameter logistic regression model using the GraphPad Prism 7 software (San Diego, CA).

**Evaluation of mitochondrial membrane potential**

To assess ΔΨ_m,i_, cells were treated with tetramethylrhodamine methyl ester (TMRM; 50 nM) for 15 min prior to analysis by flow cytometry (BD LSRFortessa, San Jose, CA, USA). Carbonyl cyanide *m*-chlorophenyl hydrazine (CCCP; 20 μM) was add 5 min before TMRM to see specific suppression of ΔΨ_m,i_. Cells without added TMRM probe were used as a control of nonspecific signal.

**ATP assays**

ATP was assessed in cells maintained in media containing 4.5 mg/L glucose and 50 μM 2-deoxyglucose (2DG) for 24 h using the CellTiter-Glo Luminescent Assay kit (Promega). The results were normalized to the total protein level evaluated in cell lysates using the bicinchoninic acid (BCA) method (Pierce Biotechnology, IL, Rockford, USA).

**Evaluation of migrastatic and anti-invasive properties of MitoTam**

Migrastatic and anti-invasive properties of MitoTam were assessed using 2- (2D) and 3-dimensional (3D) models basically as reported.^3,4^ 2D wound healing assay was performed on the confluent cell monolayer using a sterile P200 pipette tip. The released cells were washed with a pre-warmed PBS and wells were filled with a growth medium without or with added 0.625 μM MitoTam. 3D cell culture spheroids were formed in agarose mold for 48-72 h. The spheroids were embedded into collagen from rat tails (1 mg/ml) and treated with different concentrations (0-1.25 μΜ) of MitoTam.

The images were captured using a Nikon Eclipse TE2000-S microscope or Leica Thunder system equipped with LAS-X Navigator software module. Data analysis was done using ImageJ/Fiji. The spheroid area was delineated using the Threshold or Edge Finder tool. The invasion index was calculated as a normalized ratio of the spheroid area of interest to the starting spheroid area (0 h). Alternatively, the initial spheroid area was subtracted from the total spheroid area at time 48 h, and the average was normalized to the maximum value. Cell tracking was performed using the TrackMate Plugin of ImageJ/Fiji. For TrackMate, cells were highlighted by background suppression using Edge Finder and Binary Masking. Automatic cell tracking was performed on ~70 cells. The tracks were visually checked to confirm their accuracy and filtered for full track length. Manual cell tracking was done on 15 cells at each time point.

**Evaluation of respiration and glycolysis**

In most cases, respiration was assessed using the high-resolution Oxygraph O2k apparatus purchased from Oroboros (Innsbruck, Austria). Routine, complex I (CI)- and CII-dependent respiration was evaluated in cultured cells and in tissues as described.^3^ For the effect of MitoTam on tissue respiration, fresh tumor and non-tumor tissue (3-5 mg per assay) was minced in the PBI-Shredder SG3 (Oroboros), placed in the Oxygraph chamber and supplemented with increasing doses of MitoTam. Basal respiration (oxygen consumption rate, OCR) and glycolysis (assessed on the basis of its marker extracellular acidification rate, ECAR) were assessed using the Seahorse XFe96 Analyzer (Santa Clara, CA, USA) as described.^2^

**Electrophoresis and western blotting**

Cells were washed twice with PBS, harvested into Laemmli SDS sample lysis buffer (2% SDS, 50 mM Tris-Cl, 10% glycerol in double distilled H_2_O) and sonicated (2x10 s at 1 micron amplitude with 10 s cooling interval) using the Soniprep 150 instrument (MSE, London, UK). Protein concentration was estimated using the BCA method (Pierce Biotechnology). Cell lysates were supplemented with 100 mM DTT (Sigma) and 0.01% bromophenol blue (Sigma) before separation by SDS-PAGE. The same amount of protein (50-70 μg) was loaded into each well. The protein was transferred onto a nitrocellulose membrane using wet transfer and detected by specific antibodies combined with horseradish peroxidase-conjugated secondary antibodies (goat anti-rabbit or goat anti-mouse). Peroxidase activity was detected using the ECL Western Blotting Substrate or the SuperSignal West Femto Extended Duration Substrate (Thermo Fisher). The following antibodies were used: anti-γH2AX (05-636; Millipore, Billerica, MA, USA), anti-cleaved caspase-3 (9661S; Cell Signaling), anti-RIP1 (610458; Biosciences), anti-RIP pS166 (1122S; Cell Signaling), and anti-ATP5B (HPA001520; Sigma). All antibodies were diluted 1:1,000 in 2.5% non-fat milk. HPR conjugated β-actin (PA1-183-HPR; ThermoFisher) was used as a loading control. IgG-HRP anti-rabbit (170-6515) and anti-mouse (170-6516) secondary antibodies produced in goat were purchased from BioRad Laboratories (Hercules, CA, USA). Secondary antibodies were diluted 1:10,000 in 2.5% non-fat milk.

Native blue gel electrophoresis (NBGE) followed by western blotting was carried out essentially as described^2^ using the following antibodies representing individual complexes: anti-NDUFB8 (CI) (ab110242), anti-SDHA (CII) (ab137040), anti-UQCRC2 (CIII) (ab14745), anti-COX5A (CIV) (ab110262) and anti-ATP5A (CV) (ab110273) all from Abcam (Cambridge, MA, UK). Anti-HSP60 (12165; Cell Signaling) was used as loading control.

**In-gel activity**

CI, CII and CV in-gel activity assays were as described,^5^ using digitonin-solubilized mitochondria isolated from control and MitoTam-treated cells subjected to high-resolution clear native electrophoresis. To visualized the activity, gels were developed in the CI activity assay buffer composed of mitrotetrazolium blue (NTB) and NADH, CII activity assay buffer comprising NTB and phenazine methosulfate, and CV activity assay buffer containing Pb(NO_3_)_2_.

**Quantitative real time PCR (qRT-PCR)**

Total RNA was obtained using RNAzol (400 μL for a 4 cm^2^ dish; Molecular Research Center, Cincinnati, OH, USA). First strand cDNA was synthesized from 1 μg of total RNA with random hexamer primers using Revert Aid First strand cDNA Synthesis Kit (Thermo Scientific). qRT-PCR was performed using the CFX384 Touch Real-Time PCR Detection System (BioRad, Prague, Czech Republic) with 5xHOT FIREPol Evagreen qPCR Supermix GreenE dye (Solis Biodyne, Tartu, Estonia). The relative quantity of cDNA was estimated by the ΔΔCT method, data were normalized to β-actin. The following primers were purchased from Sigma: mouse CD8: forward 5´-ACT ACC AAG CCA GTG CTG CGA A-3´, reverse 5´-ATC ACA GGC GAA GTC CAA TCC G-3´; mouse IFNγ: forward 5´-CAG CAA CAG CAA GGC GAA AAA GG-3´, reverse 5´-TTT CCG CTT CCT GAG GCT GGA T-3´; mouse PD-L1: forward 5´-TGC GGA CTA CAA GCG AAT CAC G-3´, reverse 5´- CTC AGC TTC TGG ATA ACC CTC G-3´; mouse β-actin: forward 5´- CAT TGC TGA CAG GAT GCA GAA GG-3´, reverse 5´-TGC TGG AAG GTG GAC AGT GAG G-3´. Data are expressed as mean values ± SEM of a minimum of three independent experiments performed in triplicates.

**(Immuno)histochemistry**

Tumor and non-tumor tissue from animals subjected to various treatment modalities were snap-frozen immediately after resection from sacrificed animals. Tissue was then fixed overnight with 2% formaldehyde, washed extensively with PBS, incubated in DMSO for 1 h at RT and subsequently in 30% sucrose overnight at 4 °C. After this incubation tissue was incubated in increasing concentration of OCT mounting medium (20%, 50%, 70%) and frozen in 100% OCT Sigma). Cryo-sections (4 μm) were permeabilized by combination of DMSO (15 min at RT) and saponin (30 min at RT; ThermoFisher). After washing with PBS, cells were incubated in 10% FBS (diluted in PBS) for 30 min to block unspecific signals. After this step, cells were incubated with diluted primary antibody anti-CD8-FITC conjugated (ab237364, Abcam) (1:100 in PBS; overnight incubation at 4 °C). To counterstain nuclei, coverslips were mounted in Mowiol containing 4',6-diamidino-2-phenylindole (DAPI; Sigma) and viewed in a confocal microscope (Leica SP8).

### **TUNEL assay**

Murine tissues were harvested, mounted into cryo-blocks and sectioned as described above. Cryo-sections (4 μm; prepared as described above) were permeabilized by combination of DMSO (15 min in RT; Sigma) and saponin (30 min in RT; ThermoFisher). Apoptosis was detected using In Situ Cell Death Detection Kit, Fluorescein (11684795910, Roche) according to the manufacturer’s instructions. The positive and negative control tissues slides were incubated with DNAase (for 12 min) and/or TUNEL-label solution (1 h), respectively. DAPI (1 μg/mL) staining was used to visualize cell nuclei.

**Transmission electron microscopy (TEM)**

TEM was performed according to a standard protocol. In brief, cells were grown on cover-slips, fixed with 2.5% glutaraldehyde (Merck/Sigma-Aldrich) overnight, and post-fixed with 1% OsO4 (Merck/Sigma-Aldrich) made up in Sorensen’s phosphate buffer (0.1 M, pH 7.2-7.4), dehydrated in acetone series, and embedded in Epon-Durcupan (Merck/Sigma-Aldrich). Ultrathin sections (~70-90 nm) were cut, contrasted with uranyl acetate (Ladd Research Industries), and examined in the JEM-1400 FLASH transmission electron microscope (Jeol) at 80 kV. Images were captured with 2kx2k FLASH CMOS camera.

### **Targeted metabolomics analysis**

For measurement of intracellular amino acids, cells were rinsed twice with ice-cold 0.9% NaCl, scraped, washed at 15,000 g, 10 min at 4 °C and stored at -80 °C. Metabolites were extracted with ice-cold 75% acetonitrile containing internal standards (MassChrom^®^ Amino Acids and Acylcarnitines, Chromsystem), sonicated (10 times for 0.5 s), spun (12,000 x g, 10 min at 4 °C) and supernatant was filtered using Multiscreen filter plate (Merck). The levels of metabolites were estimated by hydrophilic interaction liquid chromatography coupled to QTRAP 5500 mass spectrometer (SCIEX) as described.^6^ The data were analyzed using Skyline software^6^ and processed as described.^6,8^

For measurement of intracellular nucleotides and energy charge, cells grown in a 6-well plate were washed with ice cold 0.9% NaCl solution, scraped into cold extraction buffer (80% LC-MS grade methanol in water) and stored at -80 °C. Next, proteins were pelleted by centrifugation at 20,000 x g for 15 min at 4 °C. Part of the supernatant was transferred to LC-MS vials. The cell pellet was lysed in 200 mM NaOH for 20 min at 95 °C. After centrifugation at 1,000 x g for 10 min, protein levels were measured by BCA for normalization purposes. Target measurements of nucleotides and energy balance ([ATP + ½ ADP]/[ATP+ADP+AMP]) were performed using a Dionex UltiMate 3000 LC System (Thermo Fisher Scientific) in-line connected to a Q Exactive Orbitrap mass spectrometer (Thermo Fisher Scientific). 10 µL of the sample extracts (collected in 80% LC-MS grade methanol in water as described above) were separated on an Acquity HSS T3 UPLC column (Waters Corp, 2.1 mm x 150 mm, 1.8 µm particle size) using an Ultimate 3000 HPLC (Dionex, Thermo Fisher Scientific). Column temperature was held at 40 °C throughout the analysis. Elution of metabolites was performed using a quaternary solvent system consisting of solvent A (10 mM tributylamine, 15 mM acetic acid 5% methanol in water) and solvent B (100% methanol). The flow rate was kept constant at 250 µL/min and the following linear gradient is applied: at 0 min 0% B, from 2 to 7 min an increase to 37% B is accomplished; from 7 to 14 min an increase to 41% B is carried out, from 14 to 26 min the gradient increases to 100% B and is maintained until 30 min. At 31 min the gradient progressively decreases to 0% B and kept till 40 min. The data was then collected in “centroid data acquisition” mode, with negative electrospray ionization over a mass range of *m*/*z* 70-1050 starting from min 8, both at a mass resolution of 70,000 FWHM (at *m*/*z* 200). The detector was a Q Exactive (Thermo Fisher Scientific). We used following source settings: sheath gas flow rate at 50, aux gas flow rate at 10, spray voltage at -4 kV, capillary temperature at 350 °C and the S-lens RF level was set at 60. For data processing, mass spectrometry files were converted to the mzXML format using msConvert available from the ProteoWizard toolkit.^9^ Analysis was performed using the Quan software (Thermo Fisher Scientific, Xcalibur version 4.0) and manually verified.

### **Proteomic analysis**

Cell pellets were lysed in 100 mM TEAB containing 2% SDC and boiled at 95 °C for 5 min. Protein concentration was determined using BCA protein assay kit (Thermo Fisher Scientific) and 20 µg of protein per sample was used for MS sample preparation. Cysteine residues were reduced with 5 mM final concentration of TCEP (60 °C for 60 min) and blocked with 10 mM final concentration of MMTS (10 min at RT). Samples were digested with trypsin (trypsin/protein ratio 1/30) at 37 °C overnight. Next, samples were acidified with TFA to 1% final concentration. SDC was removed by extraction to ethylacetate,^10^ and peptides were desalted using in-house made stage tips packed with C18 disks (Empore) as described.^11^

Nano reversed-phase columns (EASY-Spray column, 50 cm x 75 µm ID, PepMap C18, 2 µm particles, 100 Å pore size) were used for LC/MS analysis. Mobile phase buffer A was composed of water and 0.1% formic acid. Mobile phase B was composed of acetonitrile and 0.1% formic acid. Samples were loaded onto the trap column (C18 PepMap100, 5 μm particle size, 300 μm x 5 mm, Thermo Fisher Scientific) for 4 min at 18 μL/min, with loading buffer composed of water, 2% acetonitrile and 0.1% trifluoroacetic acid. Peptides were eluted with mobile phase B gradient from 4% to 35% B in 120 min. Eluting peptide cations were converted to gas-phase ions by electrospray ionization and analyzed on a Thermo Orbitrap Fusion (Q-OT- qIT, Thermo Fisher Scientific). Survey scans of peptide precursors from *m/z* 350 to 1400 were performed in the Orbitrap at 120K resolution (at *m/z* 200) with a 5x10^5^ ion count target. Tandem MS was performed by isolation at 1.5 Th with the quadrupole, HCD fragmentation with normalized collision energy of 30, and rapid scan MS analysis in the ion trap. The MS2 ion count target was set to 104 and the max injection time was 35 ms. Precursors with charge state 2-6 were sampled for MS2. The dynamic exclusion duration was set to 45 s with a 10 ppm tolerance around the selected precursor and its isotopes. Monoisotopic precursor selection was turned on. The instrument was run in top speed mode with 2 s cycles.^12^

All data were analyzed and quantified with the MaxQuant software (version 1.6.3.4).^13^ The false discovery rate (FDR) was set to 1% for both proteins and peptides and we specified a minimum peptide length of 7 amino acids. The Andromeda search engine was used for the MS/MS spectra search against the Human database (downloaded from Uniprot on September 2017, containing 20,142 entries). Enzyme specificity was set as C-terminal to Arg and Lys, also allowing cleavage at proline bonds and a maximum of two missed cleavages. Dithiomethylation of cysteine was selected as fixed modification and N- terminal protein acetylation and methionine oxidation as variable modifications. The “match between runs” feature of MaxQuant was used to transfer identifications to other LC-MS/MS runs based on their masses and retention time (maximum deviation 0.7 min) and this was also used in quantification experiments. Quantifications were performed with the label-free algorithm in MaxQuant.^14^

Data analysis was performed in R after loading the proteinGroups result files from MaxQuant. Proteins with less than 10% valid values were removed. Differentially expressed proteins and their false discovery rate (FDR) corrected p values were identified by the Limma package.^15^ We used gene set enrichment analysis (GSEA) as implemented in the clusterProfiler package (version 3.6.0)^16^ and gene set variation analysis (GSVA) as implemented in the GSVA R-package (version 1.26.0).^17^ Gene set analysis was performed using KEGG gene sets. GSEA and GSVA scores were calculated for sets with a minimum of 5 detected genes, all other parameters were default.

**Evaluation of blood cell count**

Immediately after mice were sacrificed, blood was aspirated using EDTA coated syringes into EDTA coated tubes. Complete blood count with differential was measured from whole blood using BC-5300 Auto Haematology Analyzer (Mindray, Shenzhen, China).

**Animal experiments**

Balb-c mice were used throughout the study as a model of syngeneic tumors as well as metastases derived from RenCa and 4T1 cells including their ρ^0^, ρ^0^ AOX and ATP5B^KO^ variants. For tumor formation, RenCa cells at 3x10^5^ in 100 μl of PBS were grafted subcutaneously and the animals were treated twice per week by intraperitoneal (i.p.) administration of MitoTam dissolved in 4% EtOH in corn oil and/or anti-PD-L1(BE0101) and/or anti PD-1 (BE0273) IgG dissolved in PBS (BioXCell, Lebanon, NH, USA) by i.p. administration using different regimens (see Results and Figures for details). Rat IgG2b (BE0090) and rat IgG2a (BE0089) isotype (BioXCell) were used as an appropriate controls. NSG mice were used for xenograft formation using human renal cancer CAKI-1 cells grafted s.c. at 10^6^ per mouse. Tumor volume was estimated based on the formula length x width x height/2.

To measure survival, Balb-c were grafted s.c. with RenCa cells (3x10^5^) and the animals were treated twice per week by intraperitoneal (i.p.) administration of MitoTam dissolved in 4% EtOH in corn oil and/or anti-PD-L1(BE0101)/ anti PD-1 (BE0273) IgG dissolved in PBS (BioXCell, Lebanon, NH, USA) in regime as described in Figures legends. Achievement of 1000 mm^3^ of tumor volume was determined as the endpoint of this study.

To form RenCa metastases, cells (as described in the Results and Figure legends) were injected intravenously (i.v.) at 10^5^ in 100 μl of PBS per animal. One day after injection were mice treated twice per week i.p. or i.v. with MitoTam (4 mg/kg) dissolved in 4% EtOH in corn oil or in saline, respectively. After 17 d, the mice were sacrificed and their lungs excised. Tissue sections were stained with H&E and metastatic loci counted.

To form 4T1 metastasis, 10^5^ cells were injected i.v. in 100 μL of PBS. After 17 days, the mice were sacrificed and their lungs and liver excised and processed for metastatic colonies detection as described.^18^

For orthotopic tumor formation, 5x10^4^ RenCa cells in 25 μL of PBS were surgically grafted in left kidney of Balb-c mice, and the animals were treated twice per week with MitoTam (4 mg/kg) dissolved in 4% EtOH in corn oil or with the excipient. At end-point, the mice were sacrificed, and both kidneys weighed.

All mice were maintained at 22 °C and 12 h/12 h light/dark regimen with food and water provided ad libitum. These experiments were performed in agreement with the Animal Protection Law of the Czech Republic and were approved by the Ethics Committee of the Institute of Molecular Genetics, Prague (permit numbers 13/2017, 114/2019, 74/2020).

**Assessment of MitoTam distribution**

Minipigs were used for Absorption, Distribution, Metabolism and Excretion (ADME) evaluation of MitoTam and its metabolites. The study was carried out according to the ICH Guidelines M3 (R2) on non-clinical safety studies for the conduct of human clinical trials and marketing authorization for pharmaceuticals, EMA/CPMP/ICH/286/1995, 2009. Minipigs are considered a suitable animal model for conducting this type of pre-clinical study. Total number of animals used in experiments was 6 females and 3 males, which was considered to be the minimum number necessary to meet scientific and regulatory guidelines for this type of study (OECD Principles). Experimental animals were treated with MitoTam at 6 mg/kg under general anesthesia. MitoTam dissolved in saline was administered by infusion via ear vein in 10 ml/kg of body weight within administration times of 20 to 36 min (mean = 26.7 min, median = 25 min). All animals were subjected to necropsy 24, 72, and 168 h after the administration of the drug.

Animals were housed in individual cages under conventional laboratory conditions. The temperature was 15-29 °C, relative humidity 20-90%. The room was monitored and ventilated. The lighting regime consisted of 13 h light/11 h dark cycles. Feeding was provided 2 times per day and water *ad libitum.* Animal care was in compliance with the European convention for the protection of vertebrate animals used for experimental and other scientific purposes (ETS 123), the Czech, Collection of Laws No. 246/1992, inclusive of the amendments, on the Protection of animals against cruelty, and Public Notice of the Ministry of Agriculture of the Czech Republic, Collection of Laws No. 419/2012 as amended, on keeping and exploitation of experimental animals. The experimental facility is a holder of the Accreditation Certificates of Ministry of Agriculture (16OZ25207/2014-17214, Act Number. 9321/2015-MZE-17214) for users issued by the Central Committee for Animal Protection of the Czech Republic. A study plan was prepared for this type of experiment and approved by the Institutional Animal Care and Use Committee (IACUC) and the Resort Professional Commission of the Czech Academy of Sciences for Approval of Projects of Experiments on Animals (Approved Protocol No. 92/2019). Procedures used in this plan were designed to conform to accepted practices and to minimize or avoid causing pain, distress, or discomfort to the animals.

Highly precise, accurate and selective method for the determination of MitoTam and its metabolites MitoTam-*N*-oxide and *N*-demethyl-MitoTam in minipig tissue, plasma and bile samples was developed. The method was fully validated for liver and plasma in compliance with the OECD Principles of Good Laboratory Practice, C (97)186/Final, Directive 2004/10/EC. Analyte separation was performed using the Thermo Fisher Scientific Vanquish UHPLC instrument with ACQUITY UPLC CSH Fluoro-Phenyl Column (2.1 x 50 mm; 1.7 μm, Waters, catalog number: 186005351). Hybrid quadrupole/orbitrap mass spectrometer Q Exactive Plus was used for detection. The samples were prepared from individual tissues (25 mg), which were ground using liquid nitrogen in mixer mill MM400 (Retsch). Ground samples were extracted with water/methanol mixture at the ratio of tissue/water/ethanol 1:9:40 (w/v/v).

**Patient data**

Patient data shown in this report are derived from the MitoTam-01 Phase 1/1b clinical trial (EudraCT 2017-004441-25). Phase 1b comprised 4 cycles (8 week duration) in bi-weekly regimen, with one week of treatment (3x1 mg/kg of MitoTam, D1, D3, D5) followed by one week of recovery. This regime was applied repeatedly. MitoTam was administered intravenously in 250 mL of physiological solution via a central catheter over 1 h. The trial was conducted over the last two years in the General University Hospital in Prague, Czech Republic, and characteristics of the two patients documented in this report are presented in Supplementary Tables I and II. Written informed consent was signed by patients.

PD-L1 protein levels in patient serum were assessed by ELISA (BMS2212; Invitrogene) according to the manufacturer´s protocol.

**Statistical analysis**

Unless stated otherwise, data are mean values ± standard error of means (SEM) of at least three independent experiments. In mouse experiments, groups of 5-10 animals were used, unless stated otherwise. One-way (to compare one main factor) or two-way ANOVA (to compare 2 main factors) followed by Tukey's multiple comparisons test or presented as mean ± SEM were used to assess statistical significance with * p<0.05, ** p<0.01, *** p<0.005 being regarded as significant, using the GraphPad Prism software. Images are representative of at least three independent experiments.

**REFERENCES**

1. Tessier PE, Penwell AJ, Souza FE, Fallis AG. [(Z)-tamoxifen and tetrasubstituted alkenes and dienes via a regio- and stereospecific three-component magnesium carbometalation palladium(0) cross-coupling strategy.](https://pubmed.ncbi.nlm.nih.gov/12916963/) *Org Lett.* 2003;5:2989-2992.
2. Bajzikova M, Kovarova J, Coelho A, et al. Reactivation of dihydroorotate dehydrogenase by respiration restores tumor growth of mitochondrial DNA-depleted cancer cells. *Cell Metab.* 2019;29:399-416.
3. Gemperle J, Dibus M, Koudelková L, Rosel D, Brábek J. [The interaction of p130Cas with PKN3 promotes malignant growth.](https://pubmed.ncbi.nlm.nih.gov/30422386/) *Mol Oncol.* 2019;13:264-289.
4. Jobe NP, Rösel D, Dvořánková B, et al. [Simultaneous blocking of IL-6 and IL-8 is sufficient to fully inhibit CAF-induced human melanoma cell invasiveness.](https://pubmed.ncbi.nlm.nih.gov/27102177/) *Histochem Cell Biol.* 2016;146:205-217.
5. Vondrusova M, Bezawork-Geleta A, Sachaphibulkij K,Truksa J, Neuzil J. The effect of mitochondrially targeted anti-cancer agents on mitochondrial (super)complexes. *Methods Mol Biol* 2015;1265:195-208.
6. Yuan M, Breitkopf SB, Yang X, Asara JM. A positive/negative ion-switching, targeted mass spectrometry-based metabolomics platform for bodily fluids, cells, and fresh and fixed tissue. *Nat Protoc.* 2012;7:872-881.
7. MacLean B, Tomazela DM, Shulman N, et al. Skyline: an open source document editor for creating and analyzing targeted proteomics experiments. *Bioinformatics.* 2010;26:966-968.
8. Dunn WB, Broadhurst D, Begley P, et al. Goodacre, Procedures for large-scale metabolic profiling of serum and plasma using gas chromatography and liquid chromatography coupled to mass spectrometry. *Nat Protoc.* 2011;6:1060-1083.
9. Chambers MC, Maclean B, Burke R, et al. [A cross-platform toolkit for mass spectrometry and proteomics.](https://pubmed.ncbi.nlm.nih.gov/23051804/) *Nat Biotechnol.* 2012;30:918-920.
10. Masuda T, Tomita M, Ishihama Y. [Phase transfer surfactant-aided trypsin digestion for membrane proteome analysis.](https://pubmed.ncbi.nlm.nih.gov/18183947/) *J Proteome Res.* 2008;7:731-740.
11. Hebert AS, Richards AL, Bailey DJ, et al. The one hour yeast proteome. *Mol Cell Proteomics.* 2014;13:339-347.
12. Rappsilber J, Mann M, Ishihama Y. [Protocol for micro-purification, enrichment, pre-fractionation and storage of peptides for proteomics using StageTips.](https://pubmed.ncbi.nlm.nih.gov/17703201/) *Nat Protoc.* 2007;2:1896-1906.
13. Cox JM, Mann M. MaxQuant enables high peptide identification rates, individualized p.p.b.-range mass accuracies and proteome-wide protein quantification. *Nat Biotechnol.* 2008;26;1367-1372.
14. Cox J, Hein MY, Luber CA, et al. Accurate proteome-wide label-free quantification by delayed normalization and maximal peptide ratio extraction, termed MaxLFQ. *Mol Cell Proteomics.* 2014;13:2513-2526.
15. Yu G, Wang LG, Han Y, He QY. ClusterProfiler: an R package for comparing biological themes among gene clusters. *Omics.* 2012;16:284-287.
16. Ritchie ME, Phipson B, Wu D, et al. Limma powers differential expression analyses for RNA-sequencing and microarray studies. *Nucleic Acids Res* 2015; 43: e47.
17. Hanzelmann S, Castelo R, Guinney J. GSVA: gene set variation analysis for microarray and RNA-seq data. *BMC Bioinformatics.* 2013;14:7.
18. Pulaski BA, Ostrand-Rosenberg S. Mouse 4T1 breast tumor model. *Curr Protoc Immunol.* 2001;Chapter 20:Unit 20 2.

**LEGENDS TO SUPPLEMENTARY FIGURES**

**FIGURE S1**

Structure of MitoTam compared to tamoxifen (A). MitoTam was injected into pigs via ear vein at 6 mg/kg, and its metabolites MitoTam-*N*-oxide (MitoTam-NOx) (B) and *N*-demethyl-MitoTam (MitoTam-DM) (C) analyzed in tissues as shown after 24, 72 and 168 h using LC-MS. MitoTam, MitoTam-DM and MitoTam-NOx were analyzed in the bile 24, 48 and 72 h after administration of MitoTam as above using LC-MS (D). Renal cancer cell lines were treated with MitoTam for 24 h as shown and viability was assessed by the Crystal violet assay (E). Data are mean values from three independent experiments ± SEM.

**FIGURE S2**

RenCa cells were seeded in a 96-well plate at 10^4^ per well and treated with 1 μM MitoTam in the presence of 50 μM Q-VD-OPh or 50 μM necrostatin-1 as shown (A) or 50 μM Q-VD-OPh alone (B), and assessed for cell death using Sytox-Green and the LS720 instrument. Parental, ρ^0^, ρ^0^ AOX and ATP5β^KO^ 4T1 cells were assessed for routine respiration using the O2k instrument (C). Parental, ρ^0^, ρ^0^ AOX and ATP5β^KO^ 4T1 cells (see panel D for WB of ATP5β wild type and knock out cells) were exposed to 1 μM MitoTam for 24 h and assessed for viability using annexin V/Hoechst 33342 and flow cytometry (E). Parental, ρ^0^, ρ^0^ AOX and ATP5β^KO^ 4T1 cells were exposed to 1 μM MitoTam for 6 h and assessed for ΔΨ_m,i_ in the absence or presence of 20 μM CCCP using TMRM and flow cytometry (F). RenCa cells were exposed to MitoTam at the concentrations shown in the absence or presence of 20 μM CCCP and assessed for ΔΨ_m,i_ using TMRM (G). Using the Seahorse XFe96 analyzer, RenCa cells treated with 0.5 μM MitoTam for 6 h were assessed for basal respiration by evaluating of the OCR (H), for glycolysis and glycolytic capacity by assessing the ECAR (I), and for ATP generation derived from the OCR measurements (J). RenCa cells were treated with 0.5 μM MitoTam for 2.5 h and assessed for the level of ATP in the absence or presence of 50 μM 2DG using a Cell-Titer Glo kit (K). RenCa cells were exposed to MitoTam at concentrations and times shown and subjected to transmission electron microscopy (L). Data are mean values ± SEM. Images are representative of three independent experiments.

**FIGURE S3**

(A) Healthy right kidney from Balb-c mice with orthotopic renal tumor in left kidney treated i.p. with MitoTam at 4 mg/kg twice per week for 2 weeks was fixed and paraffin-embedded; sections were cut (5 μm) and stained with H&E. Scanning brightfield images of kidneys show overview of the renal cortex and medulla. (B) Higher magnification images show the glomeruli and associated renal tubules in the cortex and medulla. PCT, proximal convoluted tubule; DCT, distal convoluted tubule; CD, collecting duct; TLH, thin segment of loop of Henle; TAL, thick ascending loop of Henle. Balb-c mice were grafted s.c. with 3x10^5^ RenCa cells per animal and tumors treated with 4 or 6 mg/kg MitoTam twice per week for 2 weeks. At endpoint, mice were sacrificed, and tumors sections were evaluated for proliferation using anti-Ki67 (C) and for cell death using the TUNEL assay (D) by confocal microscopy. The bar represents 100 μm. Balb-c mice were grafted s.c. with RenCa cells at 3x10^5^ per animal, treated i.p. with MitoTam as shown and assessed for their body weight (E). Balb-c mice were grafted with s.c. 4T1 cells at 10^6^ per animal and treated i.p. with MitoTam at 4 mg/kg for 2 weeks twice per week. At end point, blood was withdrawn and evaluated for blood cell count as shown (F). 3D cell culture spheroids formed from 4.5x10^3^ CAKI-1 cells were treated with MitoTam as shown, and recorded under a light microscope for 48 h. Invasion index was calculated after 48 h of treatment (left panel (**G**). Parental, ρ^0^, ρ^0^ AOX and ATP5β^KO^ 4T1 3D spheroids were prepared by plating 800 cells, treated with different concentrations of MitoTam for 48 h, and captured using a light microscope. Invasion index was calculated for each condition and cell line (**H**). Automatic mobility tracking was performed on parental cells grown under 2D conditions. The confluent cell monolayer was ‘injured’ using 200 µL pipette tip, the growth medium was changed for medium supplemented with 0.625 µM MitoTam, and wound healing was recorded at 1-h intervals for 48 h. The left panel shows light microscopy images of control and MitoTam-treated cell cultures at 0 and 48 h. The bar graph (right panel) represents the mean velocity of ≈70 cells at times 0 and 48 h (**I**). Data are mean values ± SEM. Images are representative of three independent experiments.

**FIGURE S4**

Balb-c mice were injected s.c. with RenCa cells at 3x10^5^ per animal and treated i.p. with anti-PD-L1 IgG as shown when tumors reached ≈50 mm^3^ and assessed for tumor volume (A). At endpoint, mice were sacrificed and tumors assessed for their weight (B) and for the level of CD8 (C) and IFNγ (D) mRNA using qRT-PCR. Serum obtained from blood drown from patients with clear cell renal carcinoma treated with MitoTam between cycle 1 and 8 of the therapy (C1D5 = 5^th^ day of cycle 1, C4D5 = 5^th^ day of cycle 4, C5D5 = 5^th^ day of cycle 5, C8D5 = 5^th^ day of cycle 8) and assessed for the level of PD-L1 protein using an ELISA assay (E). Data are mean values ± SEM for panels A-D, data in panel E reflect a single measurement.
