# Supplementary figures and images for: Simultaneous targeting of mitochondrial metabolism and immune checkpoints as a new strategy for renal cancer therapy
Source: Clin Transl Med. 2022 Mar 29;12(3):e645. doi: 10.1002/ctm2.645 (PMC8964933; doi:10.1002/ctm2.645)

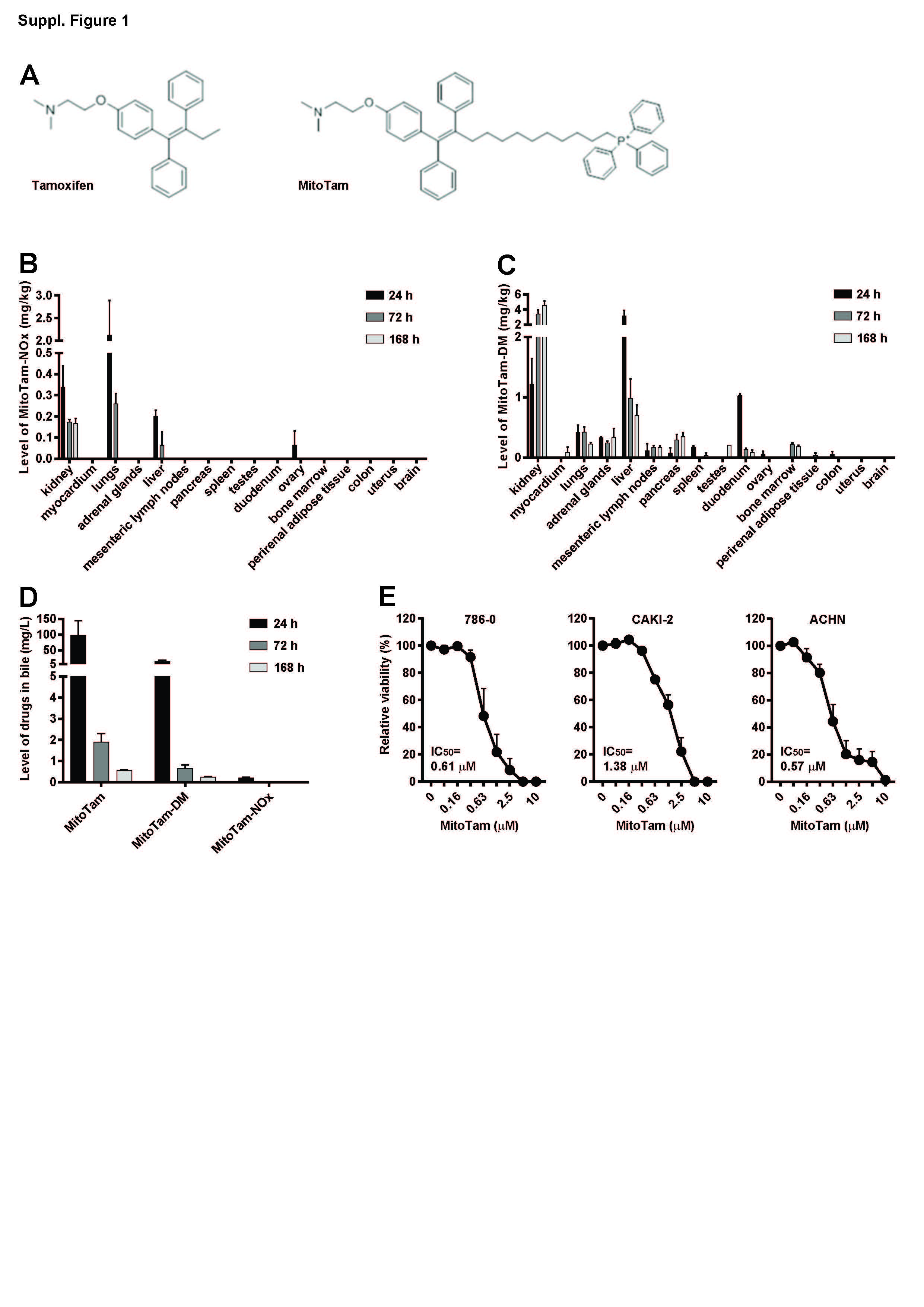

Supplement: Supplementary file 2 — Figure S1 [file CTM2-12-e645-s008.jpg]

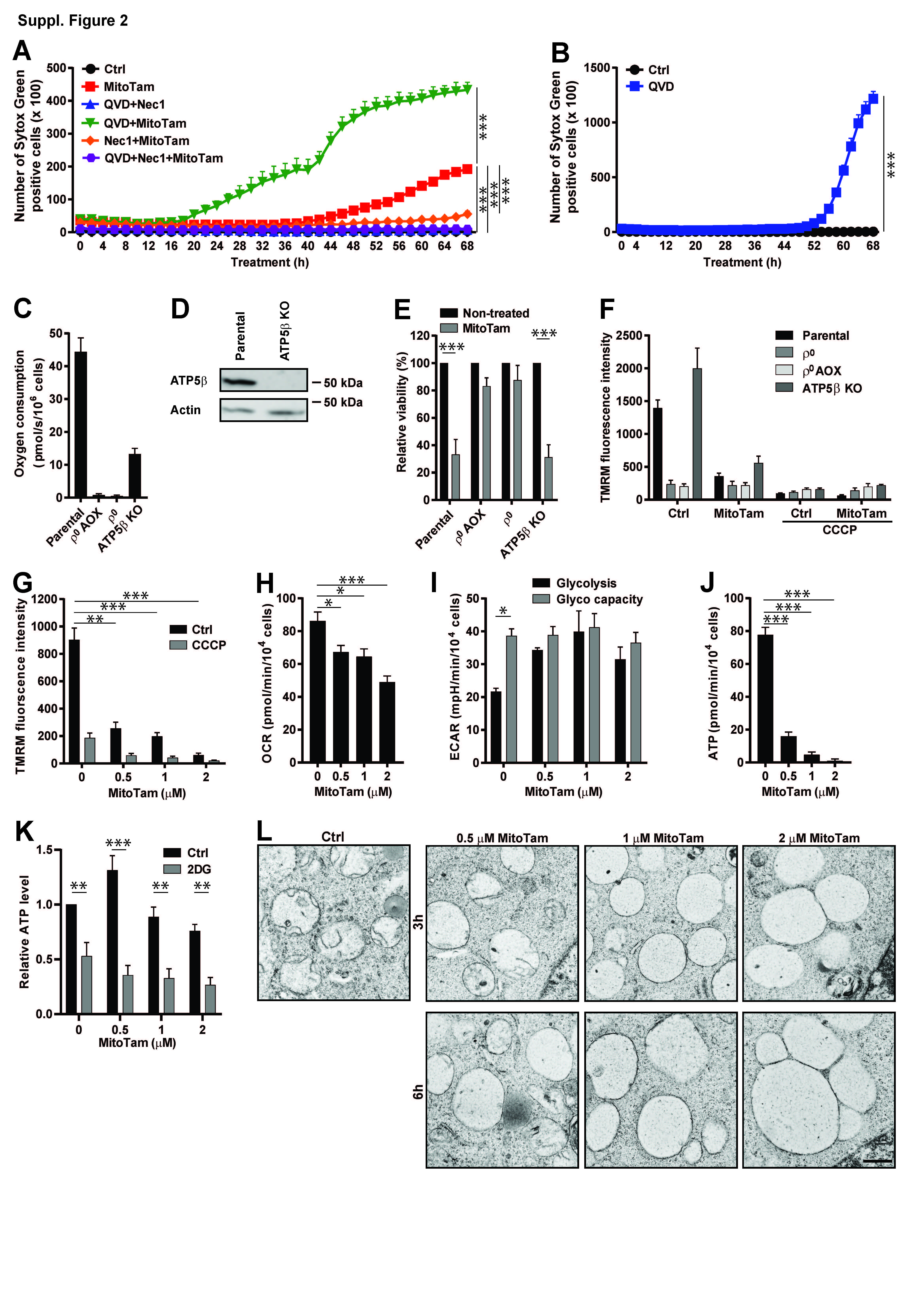

Supplement: Supplementary file 3 — Figure S2 [file CTM2-12-e645-s001.jpg]

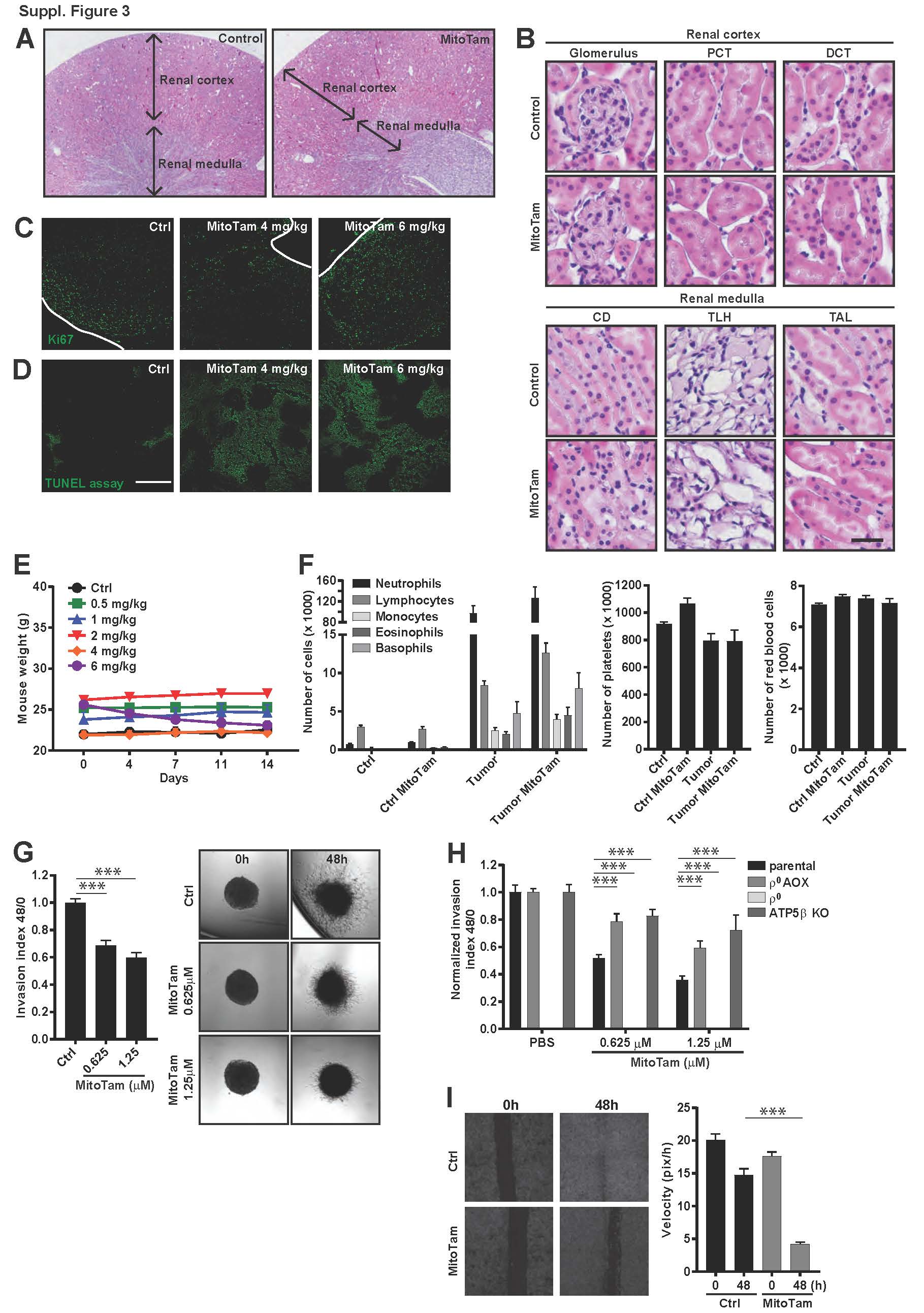

Supplement: Supplementary file 4 — Figure S3 [file CTM2-12-e645-s002.jpg]

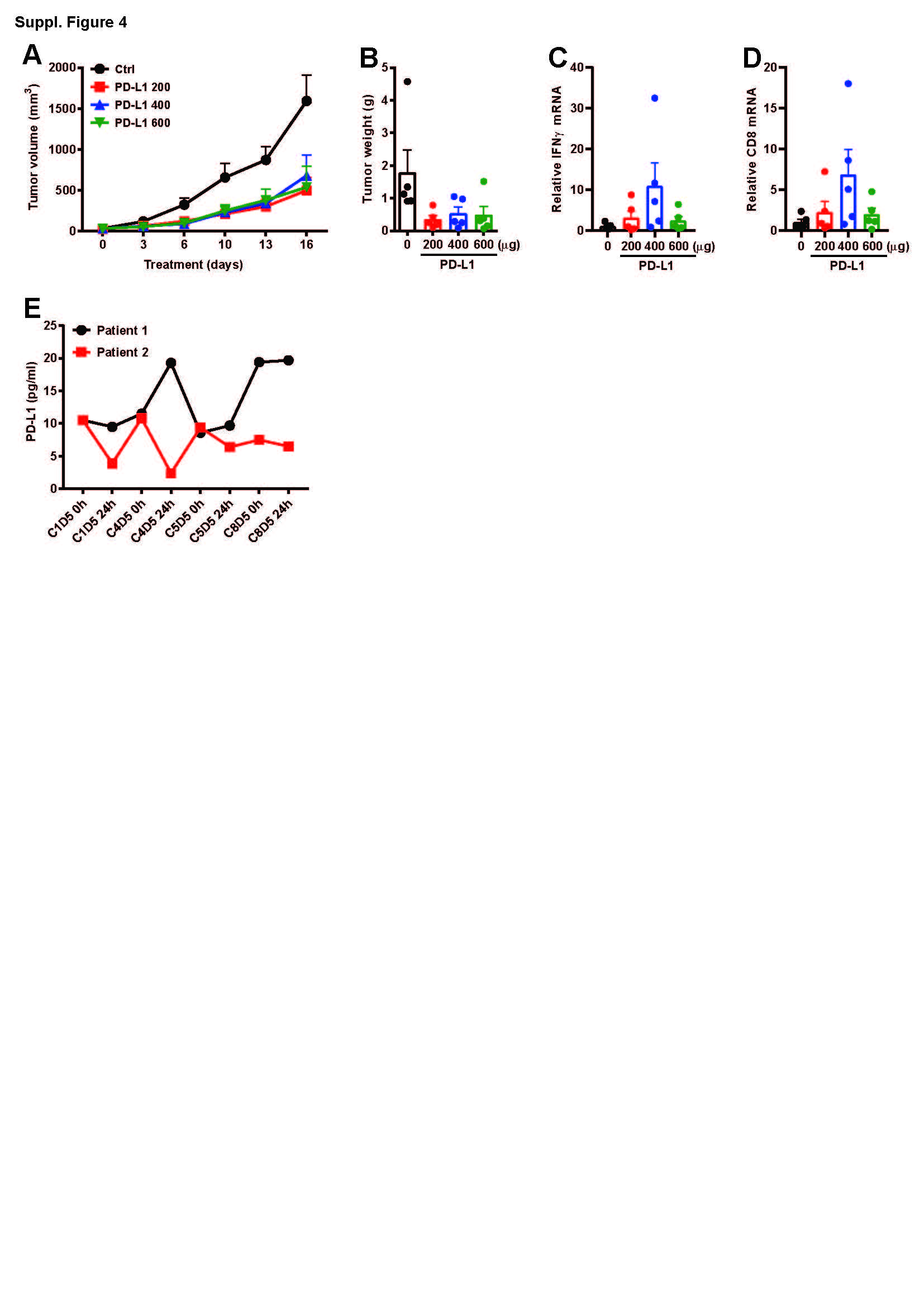

Supplement: Supplementary file 5 — Figure S4 [file CTM2-12-e645-s005.jpg]

**Table II. The size of tumors during MitoTam-01 trial**


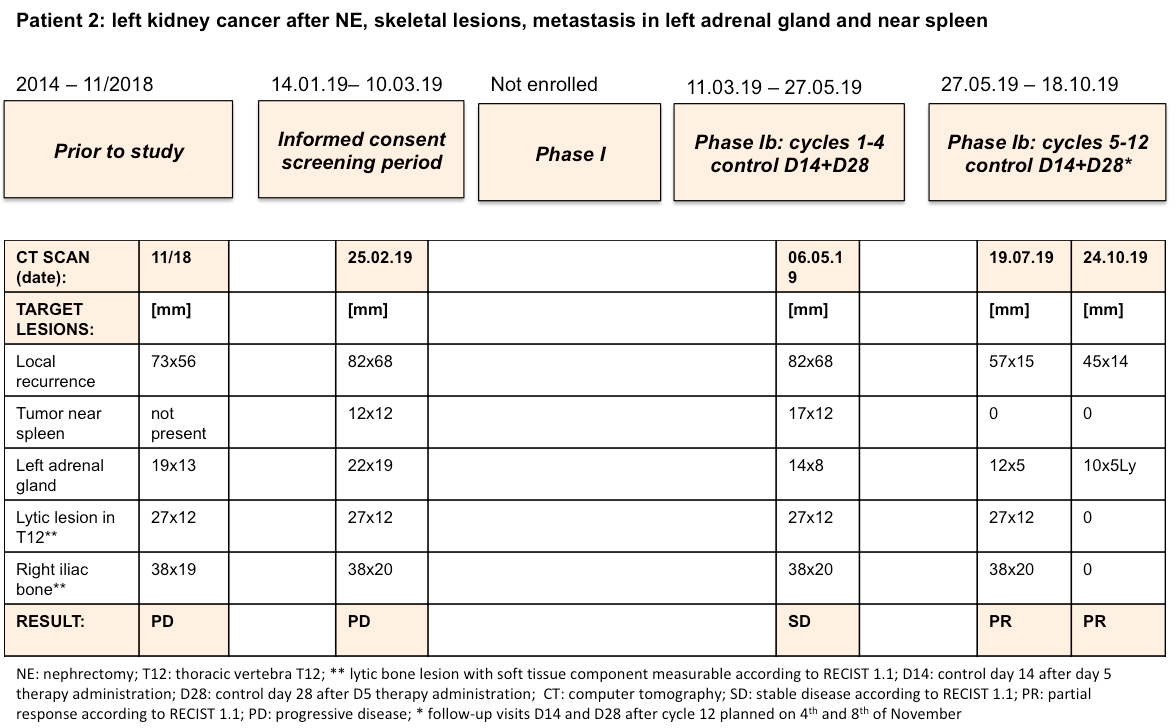


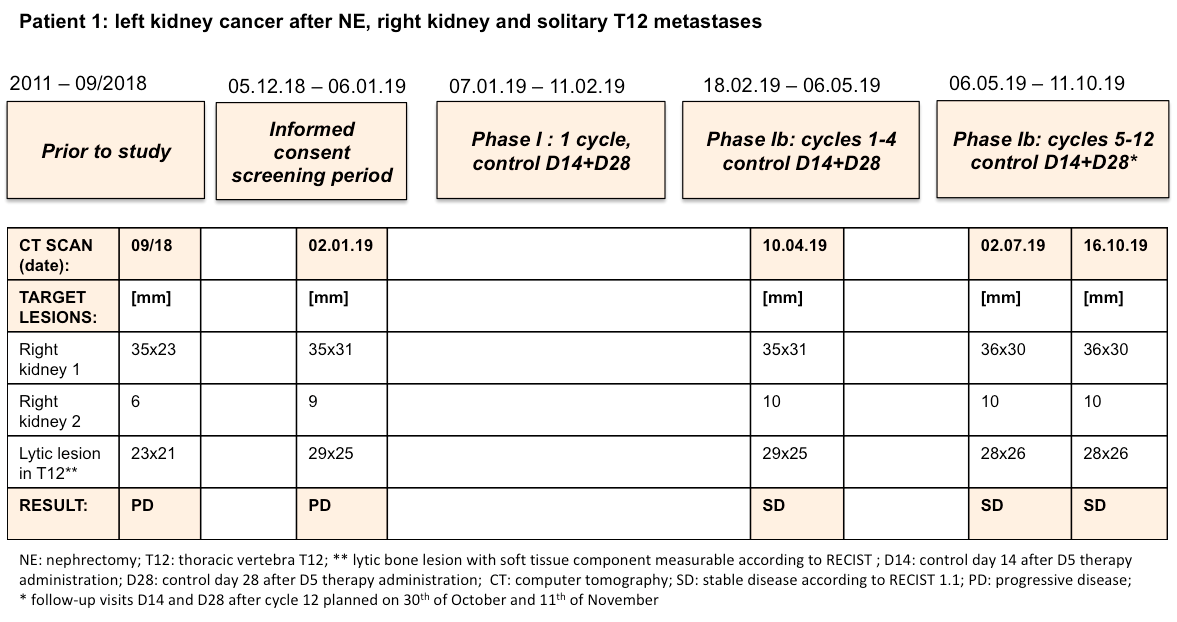

Supplement: Supplementary file 7 — Table S2 [file CTM2-12-e645-s006.docx]
