# Supplementary material for: Simultaneous targeting of mitochondrial metabolism and immune checkpoints as a new strategy for renal cancer therapy
Source: Clin Transl Med. 2022 Mar 29;12(3):e645. doi: 10.1002/ctm2.645 (PMC8964933; doi:10.1002/ctm2.645)
Supplement: Supplementary file 6 — Table S1 [file CTM2-12-e645-s004.docx]

**Table I. Patients history**

| **Patient gender** | Male | Male |
| --- | --- | --- |
| **Age (years)** | 63 | 73 |
| **Weight at screening (kg)** | 90 | 85 |
| **Comorbidities and medication** | - hypertension (perindopril/amlodipine) - hyperlipidemia (rosuvastatin) - diabetes mellitus on diet - chronic venous insufficiency (flavonoid diosminum) - hyperuricemia (alopurinol) - chronic kidney disease - prevention of SRE (denosumab, calcium, vitamin D) - thyroidectomy for metastasis of renal cancer 2011 - spontaneous pneumothorax in youth - allergy to iodine contrast agent | - skeletal pain (pregabalin) - prevention of SRE (denosumab, calcium, vitamin D) - deliberation of dural sac and T12 hemilaminectomy in 2014 because of skeletal metastases - pulmonary embolism 2017 (LMWH) - heart attack 2010 - sinusoidal bradycardia clinically nonsignificant - depressive syndrom (citalopram) - hyperuricemia (allopurinol) - chronic kidney disease |
| **ECOG at screening** | 0 | 1 |
| **Year of primary diagnosis** | 1999 (clear cells histology) | 2007 (clear cells histology) |
| **Year of disease recurrence** | 2011 | 2014 |
| **No of lines of systemic treatment** | 3 | 4 |
|  | sunitinib 03/2011-04/2012 | sunitinib 08/2014-02/2016 |
|  | everolimus 08/2012-08/2016 | axitinib 03/2016-08/2016 |
|  | sorafenib 08/2016-09/2018 | everolimus 09/2016-04/2018 |
|  |  | nivolumab 08/2018-11/2018 |
| **Palliative radiotherapy (date)** | T12 (11/2018), total dose 30 Gy | T11-L1 (09/2016) |

SRE (skeletal related event); T (thoracic vertebra); L (lumbal vertebra); LMWH (low molecular weight heparins)
